# Modulation of linguistic prediction by TDCS of the right lateral cerebellum

**DOI:** 10.1016/j.neuropsychologia.2016.04.022

**Published:** 2016-06

**Authors:** R.C. Miall, J. Antony, A. Goldsmith-Sumner, S.R. Harding, C. McGovern, J.L. Winter

**Affiliations:** a School of Psychology, University of Birmingham, Birmingham B15 2TT, UK; b Institute of Neurology, UCL, London, WC1N 3AR

**Keywords:** Cerebellum, Language, Learning, Prediction, Transcranial stimulation

## Abstract

It has been postulated recently that the cerebellum contributes the same prediction and learning functions to linguistic processing as it does towards motor control. For example, repetitive TMS over posterior-lateral cerebellum caused a significant loss in predictive language processing, as assessed by the latency of saccades to target items of spoken sentences, using the Visual World task. We aimed to assess the polarity-specific effects of cerebellar TDCS, hypothesising that cathodal TDCS should impair linguistic prediction, and anodal TDCS facilitate it. Our design also tested whether TDCS modulated associative learning in this task. A between groups (sham, anodal, cathodal) design was used, with concurrent stimulation during performance of a manual variation of the Visual World paradigm, and with assessment of latency reduction over repeated presentations of the spoken sentences. Mixed model ANOVA was used to analyse change in response latency. Cathodal TDCS decreased participants’ response time advantage for the predictable sentence items without change for non-predictable items, consistent with the previous TMS results. Furthermore, anodal stimulation enhanced the response time advantage for the predictable items, again without change in latencies for non-predictive items. We found a clear practice-based effect over 4 blocks. However, this difference was not significantly modulated by either anodal or cathodal stimulation. Our results therefore support the hypothesis that cerebellum contributes to predictive language processing, mirroring its predictive role in motor control, but we do not yet have evidence that the learning process was affected by cerebellar TDCS.

## Highlights

• Disruption of the lateral cerebellum with TMS impairs prediction in language processing.
• We now report the effect of anodal and cathodal TDCS, and assess short-term learning.
• Cathodal TDCS replicated the TMS effect, reducing linguistic prediction.
• Anodal TDCS reversed the effect, enhancing prediction; neither affected short-term learning.
• This work adds new evidence that the cerebellum contributes to predictive language processing.

## 1 Introduction

The common view that the cerebellum has a function restricted to motor control has recently shifted to accommodate evidence suggesting it also contributes to cognition. From an evolutionary perspective, the lateral cerebellum evolved following other cerebellar regions, and concurrent to the cerebral association cortex (Leiner et al., 1991). As these neocortical and neocerebellar regions evolved, neuronal connections between them also developed (Finlay and Darlington, 1995, Leiner et al., 1991). This increased cerebellar computational capacity, connecting with areas of the cerebral cortex not associated with sensorimotor processing, suggests the lateral cerebellum receives information sent from the associative and cognitive cerebral regions, and might output to the same, non-motor regions (Balsters et al., 2010, Ito, 2008, Ramnani, 2011). Evidence from functional imaging studies suggests substantial overlap in prefrontal and parietal connectivity with the posterior-lateral cerebellum, Crus II (O’Reilly et al., 2010). Thus, the more recently evolved cerebro-cerebellar pathway may function as an important - potentially bidirectional - link between the posterior and frontal lobes involved in cognitive function (Balsters et al., 2010), including language processing and inferring a cerebellar contribution to language. Consistent with this, growing evidence implicates the right lateral cerebellum in non-motor processing (Fiez, 1996, Stoodley and Schmahmann, 2009, Strick et al., 2009, Thach, 1998).

Findings from functional neuroimaging research also support the notion of right cerebellar involvement in language tasks (Desmond and Fiez, 1998, Moberget et al., 2014, Stoodley, 2011). For example, Frings et al., (2006) found greater right posterior cerebellar activity in a verb-generation task requiring participants to produce a semantically related verb in response to a noun, relative to word reading tasks, and independent of motor activity due to speech. Clinical studies on populations with right cerebellar abnormalities compliment these findings. Indeed some clinical populations showing developmental language impairments demonstrate right cerebellar irregularities; examples include cerebellar cognitive affective syndrome (Schmahmann and Sherman, 1998), smaller right cerebellar volume in language impaired individuals with autism (Hodge et al., 2010), and cerebellar structural differences in dyslexia (Eckert et al., 2003). However, clinical studies can lack specificity, because affected individuals may exhibit heterogeneous effects as well as having diverse contributing symptoms, such as working memory or attentional deficiencies.

There has been recent speculation that the cerebellar contribution to cognitive processing may have generalised from its contribution to motor control (Ramnani, 2006, Salinas et al., 2008). This is based on the uniformity of the cerebellar cortical structure, with the same internal neural circuits receiving from and targeting diverse cortical regions (Bloedel, 1992). Function may be similar across the cerebellar cortex; different cerebellar regions may equivalently manipulate signals from different cerebral regions. There is considerable evidence that the motor regions of the cerebellum form “forward models” to predict the sensory consequences of a motor response (Miall et al., 1993). These predictive estimates are learnt with experience, used to better control actions, and to anticipate sensory signals arising from action (Wolpert and Miall, 2002). The advantage of forward modelling in language processes is also recognised (Pickering et al., 2014), but evidence linking linguistic prediction to the cerebellum is still quite limited (Argyropoulos et al., 2011, Moberget et al., 2014).

Recently Lesage et al. (2012) used the Visual World Paradigm to monitor language processing through eye movements (Huettig et al., 2011) and tested the effects of cerebellar disruption with repetitive TMS. They found that low frequency rTMS over the right cerebellum increased the latency of saccades towards target images but only in conditions where a spoken verb predicted a specific target, consistent with disruption of linguistic prediction.

To our knowledge no study has yet tested both excitatory and inhibitory stimulation to enhance and disrupt cerebellar involvement in a language task. This could consolidate the argument that the cerebellum's role in language processing parallels its role in motor control. There are polarity specific effects of direct current stimulation on cerebellar excitability (Galea et al., 2009). We hypothesised, therefore, that anodal stimulation over the right cerebellum should enhance Visual World performance through exciting cerebellar activity, whilst cathodal stimulation, like low frequency rTMS, should disrupt it. In both cases, we predict that the changes would be confined to the predictive trials and not affect general performance.

There is also evidence associating cerebellar activity with practice-dependent associative learning in a verb generation task (Petersen et al., 1998) and in spoken or written word learning (Davis et al., 2009, Lesage et al., n.d.). In the motor domain, anodal cerebellar tDCS has been shown to enhance adaptation in visuomotor tasks (Galea et al., 2011), dynamic tasks (Herzfeld et al., 2014) and in sequence learning (Stagg et al., 2011), whereas cathodal stimulation or low frequency TMS worsens learning (Herzfeld et al., 2014, Jenkinson and Miall, 2010, Stagg et al., 2011). Note, however, other reports suggest less clear-cut polarity specific effects on learning (Jacobson et al., 2011, Stagg et al., 2011). Evidence also suggests the cerebellum may contribute only indirectly to learning in cognitive tasks (Pope et al., 2015). So we also hypothesised that there would be a modulation of learning over repeated blocks of the Visual World task, but we were agnostic about whether anodal or cathodal stimulation would facilitate or impair learning, compared to sham.

## 2 Methods

### 2.1 Design and participants

Seventy three healthy native English-speaking students from the University of Birmingham were sought for participation, in exchange for course credit or cash (56 were females; mean age=19.8; SD=2.7, range 18–54). All participants were screened for possible exclusion criteria for brain stimulation, including familial epilepsy, neurological medications, and recent drug, caffeine and sleep levels. The University of Birmingham ethical panel approved all procedures, and all participants gave signed, informed consent. Handedness was self-reported for 35 participants; the remainder also completed the Edinburgh Handiness scale. In total, six participants were left-handed; all participants were allowed to use the computer mouse with their preferred hand.

### 2.2 Visual world paradigm

**Fig. 1:**
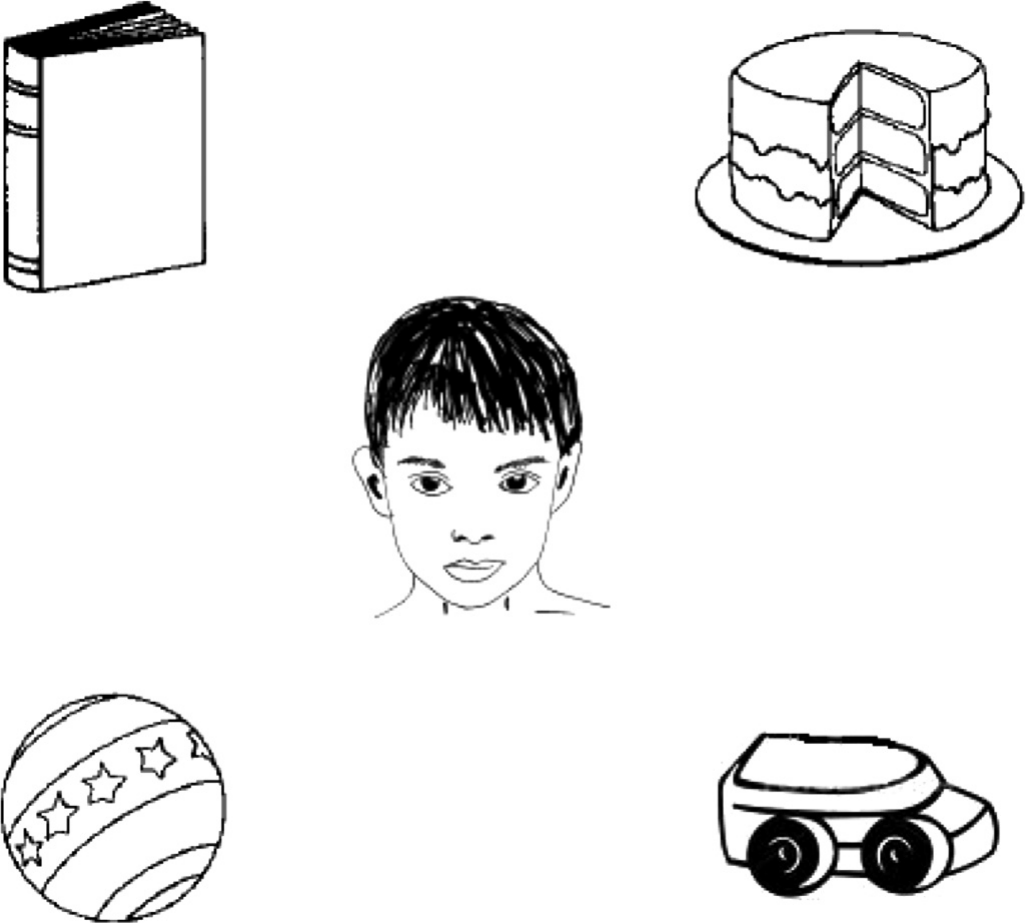
An example of a visual display in the Visual World Paradigm. In the specific condition, this static display would be presented concurrent with the spoken sentence “the boy will eat the cake”. The only plausible answer according to the specific verb “eat” is the “cake” image. In the general condition, the display might be presented along with the spoken sentence “the boy will move the cake”, or any other object (book, ball or car). Hence each of the 4 images in the display is consistent with the general verb “move”. The display screen was 25×25 cm, 820×820 pixels, and viewed at normal reading distance of approx. 50 cm.

In order to measure the speed of language processing, a manual version of the VWP was developed and run using Psychtoolbox v3 under MATLAB R2007B. On each trial the participant viewed a static visual display with one high contrast black and white line drawing image presented in each corner (Fig. 1). After 3 s a line-drawn image of a person (the agent) appeared in the screen centre to signal that the trial could begin. The participant was required to move the visible cursor onto this agent and to click the computer mouse to initiate the trial. The agent image then became low contrast, and after 500 ms a sentence spoken by either a male or female computer generated voice was delivered through headphones. The participant was required to then move the computer mouse as soon as possible towards the corner target visual image that was referenced at the end of the sentence (see legend, Fig. 1). In half the trials per block, the verb in the spoken sentence predicted one specific target image. In the other half the verb did not predict any specific target, and could be applied to all four images.

These two sentence types (specific and general) were presented pseudo-randomly. The time participants took to move the computer mouse towards the target image was measured from sentence onset, with a threshold of 200 pixels of cursor motion or about 1.5 cm of mouse movement. Short high or low beeps were automatically delivered according to correct versus incorrect responses, providing feedback to the participants.

The spoken sentences had been previously generated as.mp4 files using the “say” text-to-speech command in Mac OS X, and converted to.wav files at 44 KHz resolution. Six artificial voices were used, and for each voice, all possible sentence types were generated (64 sets of 5 sentences, one with a specific verb and object item, and four sentences with the same general verb and each of the four possible objects as the target item; 320 sentences in total for each voice).

The full experiment consisted of a practice block and five test blocks. Each block consisted of 32 trials, of which 16 used specific verbs and 16 general verbs. The order of trials within each block was generated pseudo-randomly. Each stimulus set (an agent and four object images) were displayed twice per block: on one occasion, the sentence would use a specific verb, allowing unambiguous prediction of one object. On the other occasion, the general verb would be used, and any object might be a valid target. Across the first four test blocks, each of the four objects was used as a target once, associated with the general verb, while the specific target was used four times with the same specific verb. The order of trials within each block and the location of the images in the four screen corners were pseudo-randomized. The practice block and the final test block each consisted of 32 trials with novel sets of images and sentences that were not used in the first four test blocks.

### 2.3 Transcranial direct current stimulation (tDCS)

Stimulation was administered through two sponge electrodes (surface area=5×5 cm) that were soaked in a saline solution. The direct electrical current was set to 2 mA and delivered for 20 min, using the Eldith DC-stimulator-plus by Neuroconn GmbH. One electrode was positioned 2 cm right and 1 cm below the inion (targeting the right cerebellum), while the other electrode was positioned on the right shoulder. This montage has been shown to be selective for the cerebellum (Parazzini et al., 2014). The electrode polarity was chosen to provide anodal or cathodal stimulation of the cerebellum. In each case, the current was delivered for 20 min with a 10 s ramp up and ramp down at start and end, respectively. For sham stimulation, the current ramped up and down over the first 30 s of the stimulation period. The participants performed the practice trials before the TDCS electrodes were put in place, and stimulation began 2 min before the start of the first test block of the VWP procedure. The VWP test lasted approximately 25 min.

### 2.4 Statistical analysis

The time interval between audio onset and the cursor moving more than 200 pixels towards one of the four object images was taken as the response latency.

Given that the spoken sentences were unequal in duration, both because of the word content, and also the artificial voice differences, we also measured the time interval between the stimulus onset and the final word onset, across all the stimulus sets used. This confirmed a difference in stimulus duration between the 6 voices (F(1.02,31.5)=2493, p<.0001), and a small but insignificant difference between the sentence types of 38 ms (F(1.6,50.9)=2.4, p=0.11). There was no interaction between voice and type (F(2.98,92.4)=1.1, p=0.35).

We also recorded the number of error trials. Error rates were typically below 1–3 trials per block; one participant with an overall error rate of 34% was excluded from all further analysis.

We employed mixed design ANOVAs using within-participant factors of verb type (specific or general), and block number, and a between-participant factor of tDCS stimulation type (anodal, n=26, cathodal, n=26, or sham, n=20). Greenhouse-Geisser correction for sphericity was applied to the degrees of freedom when necessary.

We first tested the omnibus null hypothesis, that response latencies were equal, with a 2 (sentence type: general, specific)×5 (block)×3 (stimulation: anodal, cathodal, sham) mixed analysis of variance (ANOVA). To examine the hypothesis that stimulation would affect response latencies for the specific sentences and would increase in effect across the experiment, as a consequence of the gradual modulation of cerebellar activity, data from blocks 1 and 5 were analysed within a 2 (sentence type: general, specific)×2 (Blocks: 1 & 5)×3 (stimulation: anodal, cathodal, sham) mixed ANOVA. Note that the stimuli used in blocks 1 and 5 were all novel to the participants, whereas the intervening blocks 2–4 repeated the stimuli from block 1, in newly randomised orders. We therefore restricted our primary analysis of the effects of TDCS to blocks 1 & 5. Significant results were followed up with further 2-way repeated measure ANOVA's between stimulation group and block, performed separately for the general and specific trials.

To next examine a learning effect, the data from the 4 repeated stimulus presentation blocks 1–4 were analysed using a 2 (sentence type: general, specific)×3 (stimulation: anodal, cathodal, sham)×4 (Block trials; 1–4) mixed ANOVA.

## 3 Results

### 3.1 Effect of sentence type

**Fig. 2:**
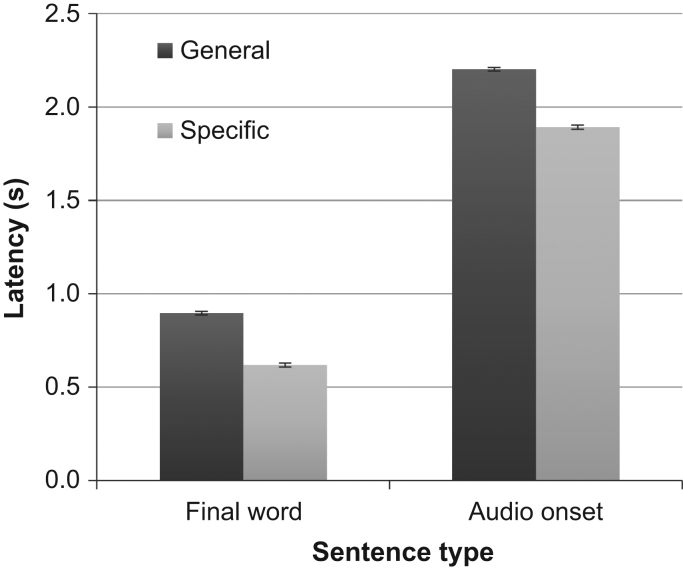
The grand mean average across all blocks and all groups for the response latency measured from final target word onset (left) or from the audio onset (right). In the general verb condition (dark grey), the latencies are 275 ms or 310 ms slower than in the specific verb condition (light grey), for the final word and audio onset measurements respectively, demonstrating the expected response latency advantage for the specific condition. Error bars are ±1 SEM.

The Visual World task is normally assessed with eye tracking to measure the difference between sentence types in saccadic latencies to fixate the target item (e.g. Lesage et al., 2012). By measuring response times with a hand-held computer mouse, we have made the task simpler to implement, but needed assurance that the main effects were similar. The omnibus ANOVA on the 72 participants showed a highly significant difference between response times for the general and specific verb trials (F(1,69)=356, p<.0001). Participants responded faster to sentences in which the spoken verb predicted a specific target image, relative to sentences in which the verb was general to all four targets, with a mean response latency difference of 310 ms (SEM=9 ms, n=360 blocks, Fig. 2). The latency advantage when measured from the onset of the final word item was 275 ms (SEM 8 ms); the 35 ms difference between these two measures is accounted for by the 38 ms difference in onset times of the general target items compared to the specific items, averaged over all sentences delivered. Thus the manual response paradigm showed the same response latency separation between general and specific verb trials as seen in ocular recordings (Altmann and Kamide, 1999).

### 3.2 Error

**Fig. 3:**
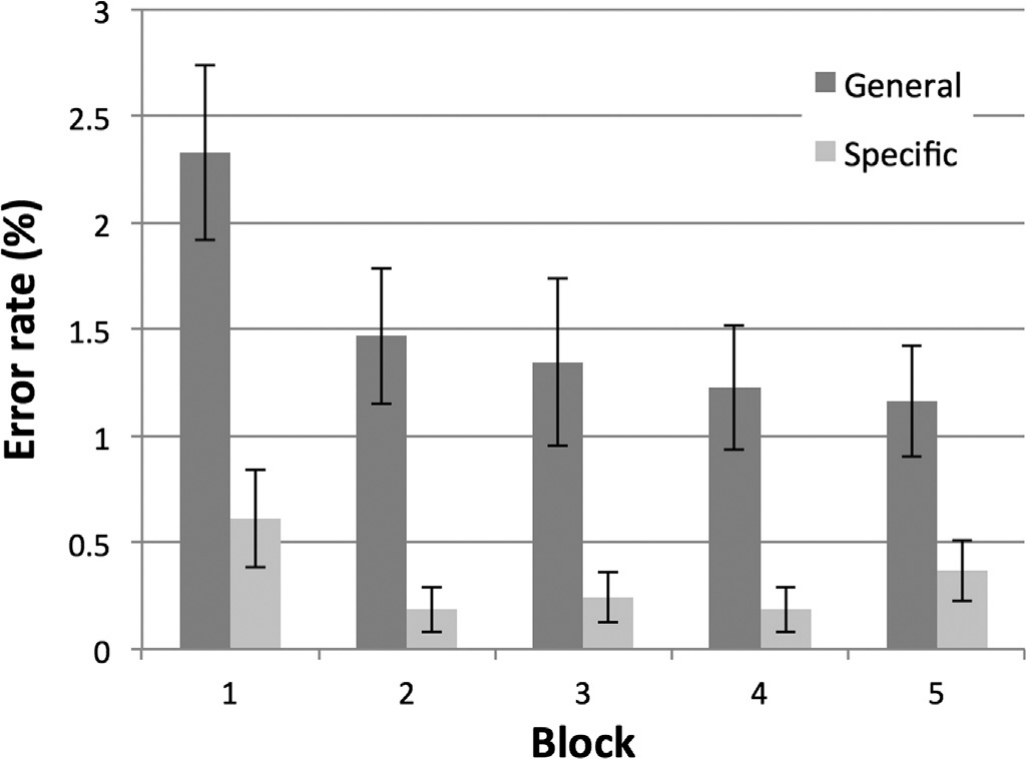
The error rate (% of trials) averaged across all participants for the general (dark grey) and specific verb trials (light grey). There was a gradual decline in errors across the 5 blocks of experiment, and significantly fewer errors in the specific condition, but no effect of stimulation condition. Error bars are ±1SEM.

Error rates, after exclusion of one participant from all analysis, fell across the 5 blocks, and were smaller for the specific condition (Fig. 3). A 2×3×5 mixed ANOVA showed the difference in errors across sentence types was significant (F(1,48)=34.4, p<.001), as was the fall across blocks (F(3.23,155.2)=3.55, p=0.014), with Greenhouse-Geisser adjusted DFs to account for significant sphericity). There was no main effect of TDCS stimulation on errors (p=0.7), nor any interaction with other factors (p>.23). Thus errors reduced with practice, and were smaller for the specific condition, as expected because of the advantage of there being a single, predictable, target item for specific trials compared with a choice of four items for the general trials.

### 3.3 Stimulation effects

**Fig. 4:**
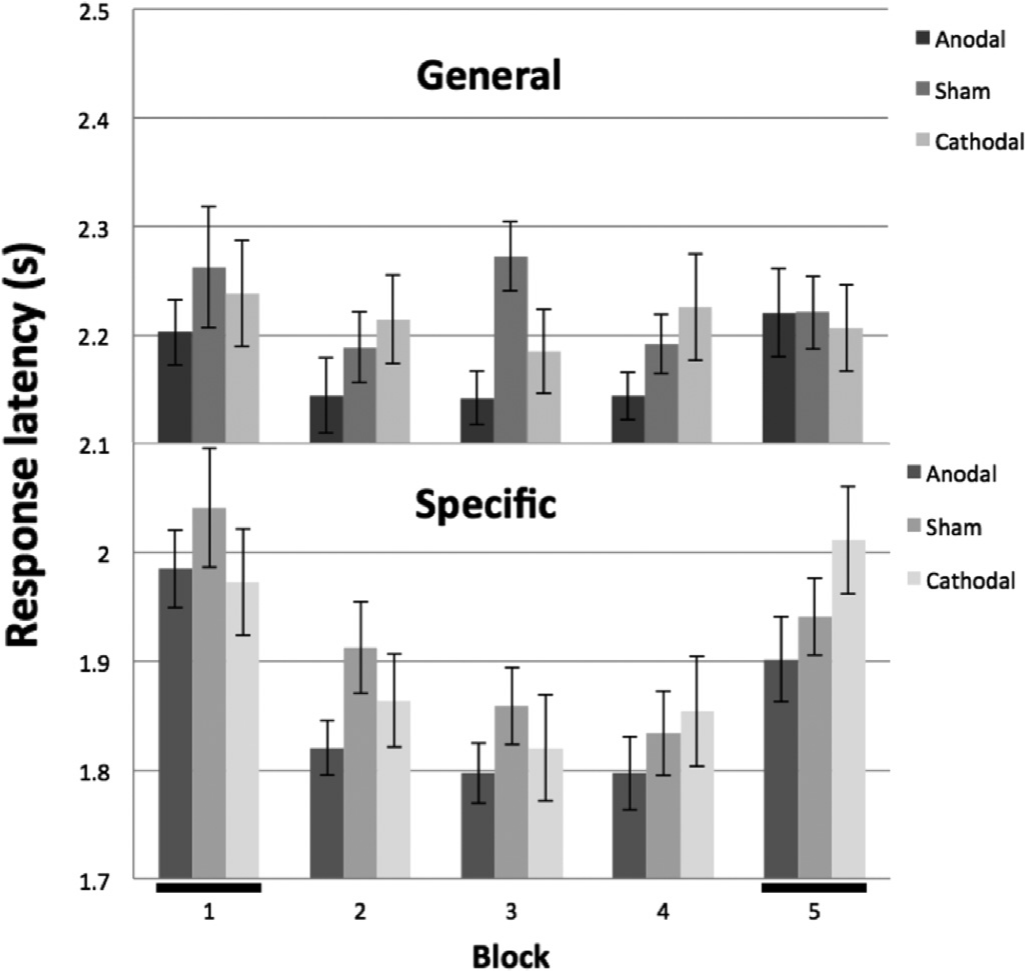
The average response latency from audio onset for the general verb trials (top) and specific verb trials (bottom) across the 5 blocks of the experiment, for the three TDCS groups. Novel stimuli were presented in Blocks 1 and 5 (black bars); Blocks 1–4 repeat the same specific verb trials, allowing learning. Error bars are ±1 SEM.

We predicted that any changes in response latency should be restricted to the specific verb conditions, both because we hypothesised that TDCS should only affect the predictive process and not affect general performance (Lesage et al., 2012) and because it was only in the specific conditions that participants were able to learn across the repeated presentations of Blocks 1–4. We therefore followed the omnibus ANOVA with separate 3 (stimulation type) x 5 (block) mixed ANOVAs for the two sentence conditions. Response latencies in the general verb condition were not affected by block (F(3.5,239.0)=2.34, p=0.065) or by stimulus condition (F(1,69)=0.88, p=0.4), nor was their interaction significant (F(6.9,239.0)=1.64, p=0.126; Fig. 4 top). In contrast, the response latencies in the specific verb condition showed a significant effect of block (F(3.6,250.5)=39.4, p<.0001; Fig. 4 bottom), with a gradual reduction in latency across Blocks 1–4 and a rebound in Block 5. While there was no main effect of stimulus group (F(1,69)=0.66, p=0.5), there was a significant interaction between block and group (F(7.3,250.5)=2.13, p=0.039). Thus, as hypothesised, there was a learning related change for the specific condition as well as an effect of TDCS that was restricted to the responses in the specific condition.

**Fig. 5:**
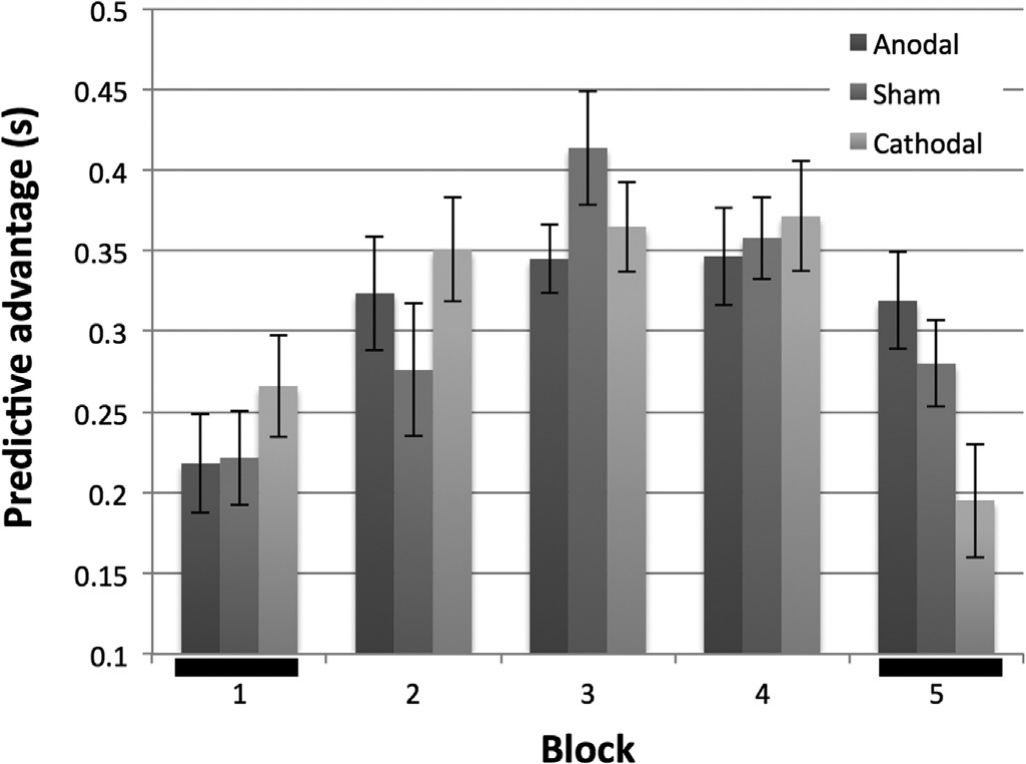
The predictive advantage (general verb response latency minus specific verb response latency) across the 5 blocks of the experiment, for the three TDCS groups. Novel stimuli are presented in Block 1 and 5 (black bars); Blocks 1–4 repeat the same specific verb trials, allowing learning. Error bars are ±1 SEM.

To explore the effect of TDCS on response latencies, we next examined the responses for only Blocks 1 and 5, in which novel stimuli were presented to the participants at the start and end of the stimulation period respectively. The 2×3×2 mixed ANOVA showed the expected main effect of sentence type (F(1,69)=356, p<.0001) and a block effect (F(1,69)=4.06, p=0.048). There was no main effect of stimulus group, but a significant interaction between sentence type, block and stimulation group (F(2,69)=4.53, p=0.014). Fig. 5 shows the difference in response latency between general specific trials, i.e. the predictive advantage across the experiment. Comparing Blocks 1 and 5 (Fig. 5 black bars) which each presented novel stimuli, it is clear that the predictive advantage increased for the anodal group (paired *t*-test, p=0.017), increased somewhat less for sham than anodal, and actually reduced for the cathodal group. The differences between the three stimulation condition groups were not significant in Block 1 (1-way ANOVA, p=0.47), whereas in Block 5 the differences became significant (F(2,69)=4.25, p=0.018), and were driven by the substantial 124 ms difference in predictive advantage between the anodal and cathodal groups (independent samples *t*-test, p=0.006).

Because left-handed individuals may have differences in language lateralization, we tested if adding handedness as a covariate affected these results; however, the interaction between sentence type, block and stimulation group remained significant (F(2,69)=4.08, p=0.021).

### 3.4 Learning effects

Finally, to explore the effect of cerebellar stimulation on learning, we compared the difference between general and specific trial response latencies across Blocks 1–4, the four blocks which used repeated presentation of the specific verb sentences (Fig. 4). In a 2×3×4 mixed ANOVA, we found the expected main effect of sentence type (F(1,69)=993 p<.0001), and of block (F(3.33,229.9)=21.5, p<.0001), and their interaction (F(3.54,244.0)=11.6, p<.0001). There was a trend for an earlier and greater reduction in response latencies in the predictive trials for the anodal group between Block 1 and 4 (187 ms, SEM 29 ms; Fig. 4 bottom) compared to cathodal group (118 ms, SEM 36 ms). However, the ANOVA found there was no significant effect of stimulus group (p=0.56) nor interactions with block (p=0.093) or sentence type (p=0.106). Hence we found no strong evidence of TDCS modulating the learning effect.

## 4 Discussion

We sought to add further weight to the accumulating evidence for a cerebellar contribution to language processing, by demonstrating a dichotomous effect of anodal and cathodal TDCS stimulation on performance within a manual response Visual World task. Consistent with the previously reported effects of repetitive TMS over the right lateral cerebellum (Lesage et al., 2012), cathodal stimulation over the right cerebellum appeared to degrade the predictive advantage in response latency for trials with specific verb sentences, while having no influence on responses to general verb sentences. Furthermore anodal TDCS facilitated responses in the predictive trials, such that by Block 5 there was a 124 ms mean difference in predictive advantage between the anodal and cathodal groups, while the sham group showed an intermediate level of performance. There was also a very clear practice-dependent learning effect for specific verb sentences over the four repeated blocks. No learning was expected for the general verb sentences, as a different display item was selected as the target amongst the sets of stimuli repeated in each block. While there was a trend for learning to be mediated by stimulation, with faster learning in the anodal group, and slower learning in the cathodal group, these effects were not statistically significant.

The Visual World Paradigm is frequently employed to examine predictive processes in language (Huettig, 2015). In this study, we have verified that the differences in manual response latencies between the two sentence conditions are consistent with those reported for eye fixations, with responses 275 ms faster for images predictable from the specific verb compared to those for images not predictable from the general verb. Altmann and Kamide (1999) reported a difference in eye fixation latencies of 227 ms between these conditions. The mean latencies of manual responses after the final word onset in our study (895 ms and 618 ms in the general and specific trials) are about 700–750 higher than the first saccade latencies that Altmann and Kamide (1999) report of approx. 127 and −85 ms, respectively, with the latter being anticipatory. Thus, we cannot claim that the manual responses are anticipatory, i.e. that they are initiated before the onset of the final word; but it is highly likely that the target image was fixated by the eye prior to the arm movement, and so we expect eye movement latencies might well be in the same range as reported by Altmann and Kamide (1999).

One limitation of the present study is that we have not contrasted the effects of stimulation over the cerebellum with that of another site. One might argue then, that extra-cerebellar systems might be involved. However, simulation studies have shown that the electrode montage used is selective for the cerebellum, with about twice the electric field amplitude than seen in occipital cortex, pons, medulla ad midbrain (Parazzini et al., 2014); moreover, the fields generated within the cerebellum are not critically dependent on electrode position (Parazzini et al., 2014). Finally, direct tests of the excitability of visual cortex and brainstem rule out inadvertent activation of occipital cortex (Galea et al., 2011) and brainstem (Galea et al., 2009) after TDCS over the cerebellum. Given the associated behavioural results in the visual world task using the more focal technique of TMS (Lesage et al., 2012), we argue that the most likely site affected by the TDCS is within the cerebellum, and that the effects are not caused by activation of more distant, non-cerebellar, sites.

The mechanism by which the cerebellum contributes to language processing prediction is unclear. One possible account is discussed by Pickering and Garrod, 2013, Pickering and Garrod, 2007 who propose that individuals exploit speech production pathways to aid comprehension. As individuals process speech, these production mechanisms are engaged to generate an internal corollary discharge of the external heard content. We suggest that the cerebellum may receive an efferent copy of this inner speech command. It then uses a forward model (which we and others propose is a function of the cerebellum; Miall et al., 1993, Wolpert et al., 1998) to predict what they are likely to articulate and hear next as inner speech (Pickering and Garrod, 2013). This prediction may be potentially based upon a simulation route (what would the listener produce under the same situation?), an associative route (what have others articulated in similar situations?) and/or on background contextual information (e.g., what are the visual display items options?). This predictive explanation complements speculations that the cerebellar contribution to language processing parallels its role in motor control (Lesage et al., 2012, Moberget et al., 2014), with both incorporating forward models.

We cannot yet know the content of the predictions. It is tempting to speculate based upon neuroanatomical connections between the cerebral language areas and the lateral cerebellum, although these also cannot yet constrain the choices. Studies in non-human primates have identified parallel cortico-cerebellar loops connecting lobule HVII (Crus I/II) with the homologue of Brodmann's area 9/46, with posterior parietal cortices and with pre-SMA (Bostan et al., 2013; Kelly and Strick, 2003; Middleton and Strick, 1998). These connections have also been reported in humans, based on resting-state functional connectivity: Crus I/II activity is correlated with areas including inferior frontal gyrus, posterior parietal cortex, and anterior cingulate cortex (Bernard et al., 2012, Buckner et al., 2011a, Habas et al., 2009). Meta-analysis also suggests the co-activation of Crus I/II with prefrontal and parietal cortices in emotion and cognition tasks (Balsters et al., 2010).

In our study, we assume that predictions were made based on the semantic content conveyed in the verb, perhaps processed by the middle temporal gyrus, which is involved in discerning the semantic content of auditory information (Hickok and Poeppel, 2007). The middle temporal gyrus may then send a copy of the semantic content to the cerebellum to which it is connected (Buckner et al., 2011b). A more direct motoric route might be possible, with an efferent copy of inner speech arising from inferior frontal gyrus, from which a forward model within the cerebellum then makes sensory predictions. What information the cerebellum receives, whether the semantic information of the speaker's intent or the internal copy of inner speech, and what it predicts, cannot yet be determined. Moreover, there are other theories suggesting how language predictions are made (reviewed in Huettig, 2015). Therefore, current findings illustrate that the cerebellum may contribute to language processing prediction, but the question remains how (Mariën et al., 2013).

Initially, by comparison with the depressive effects of rTMS previously employed (Lesage et al., 2012), it seemed surprising we found cathodal stimulation did not significantly disrupt participant's performance compared to sham (Fig. 4) whereas the main effect appears to be driven by the anodal group. A recent meta-analytical review of cognitive and motor TDCS studies (Jacobson et al., 2011) found that the likelihood of obtaining significant effects using cathodal stimulation for cognitive tasks was consistently lower than for anodal stimulation – and this polarity difference was greatest for language studies. However, responses towards the novel predictable items in Block 5 were on average slower for the cathodal group than in Block 1 (Fig. 4 bottom), whereas the anodal and sham groups showed faster responses. The predictive advantage for the specific verb trials fell by 70.9 ms from Block 1 to Block 5 for the cathodal group (Fig. 5), implying cathodal stimulation of the cerebellum may indeed block prediction, and to a degree equivalent to that seen after low frequency rTMS (Lesage et al., 2012). In contrast, the predictive advantage increased by 58.4 ms for the sham group and by 100.1 ms for the anodal group. We speculate that the sham effect reflects a general improvement in task performance due to experience, while the anodal group showed both this general effect as well as an additional facilitation of prediction driven by the cerebellar excitation, and the cathodal group showed disruption in prediction, despite increased experience in the task.

One might predict that the effects of TDCS seen between blocks 1 & 5 should also be evident in the intervening blocks 2–4, since there was a clear predictive advantage in each block for the specific trials compared to the general trials. This was not borne out however (Fig. 5). There may be two alternative explanations for this. First, there is evidence that the effects of TDCS are greatest when the task is most challenging (Berryhill, 2012, Kwon et al., 2015, Pope et al., 2015, Pope et al., 2012); given that the repetition of the specific trials in blocks 1–4 allowed a clear improvement in error rates (Fig. 3) and in response latencies (Fig. 4), regardless of stimulus modality, then it is possible that the ease of the task reduced the exposure of any TDCS effect. Second, and related, learning may engage other circuits, so that the effects of cerebellar TDCS are no longer so apparent. For example, Petersen et al., (1998) found that during the initial stages of a verb generation task, regions including the right cerebellum were involved. However, with repeated trials these regions became less active whilst other areas, notably the insula, took over. After introducing novel nouns, they found that the regions initially involved, including the right cerebellum, contributed once again. This may explain the current study's trend that the stimulation effects in the repeated Blocks 1–4 were small and not significant. Instead a build-up of the TDCS neuromodulation over the course of the 25-min experiment (Nitsche and Paulus, 2000) was behaviourally expressed during the novel trials in Block 5. The lack of stimulation effect in the repeated blocks should not be considered as evidence against cerebellar involvement in practice-dependent learning in language prediction.

Recently we have shown that cathodal cerebellar tDCS enhanced participants’ performance in a difficult mental subtraction task (Pope et al., 2012). In that study, we also showed enhanced verb generation performance after cathodal stimulation; anodal stimulation did not affect either task. Therefore, inhibitory cathodal stimulation may inhibit cerebellar Purkinje cells, resulting in neocortical regions receiving less tonic inhibition by the cerebellum. This implies opposite effects to those seen here, but only during high task demands. However, the mental subtraction task is probably very heavily dependent on frontal cortex, and is not obviously based in prediction, and we have subsequently shown similar mental subtraction task performance changes by anodal TDCS over DLPFC (Pope et al., 2015). Hence, one must be careful to separate effects due to direct influence on cerebellar processing from indirect effects on remote areas. We speculate that the cognitive facilitation seen following cerebellar cathodal stimulation (Pope et al., 2012) might be expressed if the cognitive task demands of the Visual World Paradigm were made stronger, for example by having more than 4 target choices in the display; further work will be required to test this.

Overall, our findings support the growing evidence for the cerebellum playing a role in predictive processing of spoken language (Lesage et al., 2012, Moberget et al., 2014, Pickering and Clark, 2014) consistent with its predictive role as a forward model for motor control (Wolpert and Miall, 2002). How it does this, and on the basis of what input signals remains to be answered. Importantly we have shown both facilitation and suppression of the predictive effect, without changing task performance in the non-predictive control conditions, implying a central role for the cerebellum in linguistic prediction.

## References

[bib1] Altmann G.T., Kamide Y. (1999). Incremental interpretation at verbs: restricting the domain of subsequent reference. Cognition.

[bib2] Argyropoulos G.P., Kimiskidis V.K., Papagiannopoulos S. (2011). θ-burst stimulation of the right neocerebellar vermis selectively disrupts the practice-induced acceleration of lexical decisions. Behav. Neurosci..

[bib3] Balsters J.H., Cussans E., Diedrichsen J., Phillips K.A., Preuss T.M., Rilling J.K., Ramnani N. (2010). Evolution of the cerebellar cortex: The selective expansion of prefrontal-projecting cerebellar lobules. Neuroimage.

[bib4] Bernard J.A., Seidler R.D., Hassevoort K.M., Benson B.L., Welsh R.C., Wiggins J.L., Jaeggi S.M., Buschkuehl M., Monk C.S., Jonides J., Peltier S.J. (2012). Resting state cortico-cerebellar functional connectivity networks: a comparison of anatomical and self-organizing map approaches. Front. Neuroanat..

[bib5] Berryhill M.E. (2012). Parietal contributions to visual working memory depend on task difficulty. Front. Psychiatry.

[bib6] Bloedel J.R. (1992). Functional heterogeneity with structural homogeneity: how does the cerebellum operate?. Behav. Brain Sci..

[bib103] Bostan A.C., Dum R.P., Strick P.L. (2013). Cerebellar networks with the cerebral cortex and basal ganglia. Trends Cogn. Sci..

[bib7] Buckner R.L., Krienen F.M., Castellanos A., Diaz J.C., Yeo B.T.T. (2011). The organization of the human cerebellum estimated by intrinsic functional connectivity. J. Neurophys..

[bib8] Buckner R.L., Krienen F.M., Castellanos A., Diaz J.C., Yeo B.T.T. (2011). The organization of the human cerebellum estimated by intrinsic functional connectivity. J. Neurophys..

[bib9] Davis M.H., Di Betta A.M., Macdonald M.J.E., Gaskell M.G. (2009). Learning and consolidation of novel spoken words. J. Cogn. Neurosci..

[bib10] Desmond J.E., Fiez J.A. (1998). Neuroimaging studies of the cerebellum: language, learning and memory. Trends Cogn. Sci..

[bib11] Eckert M.A., Leonard C.M., Richards T.L., Aylward E.H., Thomson J., Berninger V.W. (2003). Anatomical correlates of dyslexia: frontal and cerebellar findings. Brain.

[bib12] Fiez J.A. (1996). Cerebellar contributions to cognition. Neuron.

[bib13] Finlay B.L., Darlington R.B. (1995). Linked regularities in the development and evolution of mammalian brains. Science.

[bib14] Frings M., Dimitrova A., Schorn C.F., Elles H.-G., Hein-Kropp C., Gizewski E.R., Diener H.C., Timmann D. (2006). Cerebellar involvement in verb generation: an fMRI study. Neurosci. Lett..

[bib15] Galea J.M., Jayaram G., Ajagbe L., Celnik P. (2009). Modulation of cerebellar excitability by polarity-specific noninvasive direct current stimulation. J. Neurosci..

[bib16] Galea J.M., Vazquez A., Pasricha N., Orban de Xivry J.J., Celnik P. (2011). Dissociating the roles of the cerebellum and motor cortex during adaptive learning: the motor cortex retains what the cerebellum learns. Cereb. Cortex.

[bib17] Habas C., Kamdar N., Nguyen D., Prater K., Beckmann C.F., Menon V., Greicius M.D. (2009). Distinct cerebellar contributions to intrinsic connectivity networks. J. Neurosci..

[bib18] Herzfeld D.J., Pastor D., Haith A.M., Rossetti Y., Shadmehr R., O’Shea J. (2014). Contributions of the cerebellum and the motor cortex to acquisition and retention of motor memories. Neuroimage.

[bib19] Hickok G., Poeppel D. (2007). The cortical organization of speech processing. Nat. Rev. Neurosci..

[bib20] Hodge S.M., Makris N., Kennedy D.N., Caviness V.S., Howard J., McGrath L., Steele S., Frazier J.A., Tager-Flusberg H., Harris G.J. (2010). Cerebellum, language, and cognition in autism and specific language impairment. J. Autism Dev. Disord..

[bib21] Huettig F. (2015). Four central questions about prediction in language processing. Brain Res..

[bib22] Huettig F., Rommers J., Meyer A.S. (2011). Using the visual world paradigm to study language processing: a review and critical evaluation. Actpsy.

[bib23] Ito M. (2008). Opinion – control of mental activities by internal models in the cerebellum. Nat. Rev. Neurosci..

[bib24] Jacobson L., Koslowsky M., Lavidor M. (2011). tDCS polarity effects in motor and cognitive domains: a meta-analytical review. Exp. Brain Res..

[bib25] Jenkinson N., Miall R.C. (2010). Disruption of saccadic adaptation with repetitive transcranial magnetic stimulation of the posterior cerebellum in humans. Cerebellum.

[bib102] Kelly R.M., Strick P.L. (2003). Cerebellar loops with motor cortex and prefrontal cortex of a nonhuman primate. J. Neurosci..

[bib26] Kwon Y.H., Kang K.W., Son S.M., Lee N.K. (2015). Is effect of transcranial direct current stimulation on visuomotor coordination dependent on task difficulty?. Neural Regen. Res..

[bib27] Leiner H.C., Leiner A.L., Dow R.S. (1991). The human cerebro-cerebellar system: its computing, cognitive, and language skills. Behav. Brain Res..

[bib28] Lesage E., Morgan B.E., Olson A.C., Meyer A.S., Miall R.C. (2012). Cerebellar rTMS disrupts predictive language processing. Curr. Biol..

[bib29] Lesage, E., Nailer, E.L., Miall, R.C., n.d. Cerebellar BOLD signal during the acquisition of a new lexicon predicts its early consolidation. Brain and Language. 10.1016/j.bandl.2015.07.005 PMC5066914 26303580

[bib30] Mariën P., Ackermann H., Adamaszek M., Barwood C.H.S., Beaton A., Desmond J., De Witte E., Fawcett A.J., Hertrich I., Küper M., Leggio M., Marvel C., Molinari M., Murdoch B.E., Nicolson R.I., Schmahmann J.D., Stoodley C.J., Barwood M., Timmann D., Wouters E., Ziegler W. (2013). Consensus paper: language and the cerebellum: an ongoing enigma. Cerebellum.

[bib31] Miall R.C., Weir D.J., Wolpert D.M., Stein J.F. (1993). Is the cerebellum a smith predictor?. J. Mot. Behav..

[bib101] Middleton F.A., Strick P.L. (1998). Cerebellar output: motor and cognitive channels. Trends Cogn. Sci..

[bib32] Moberget T., Gullesen E.H., Andersson S., Ivry R.B., Endestad T. (2014). Generalized role for the cerebellum in encoding internal models: evidence from semantic processing. J. Neurosci..

[bib33] Nitsche M.A., Paulus W. (2000). Excitability changes induced in the human motor cortex by weak transcranial direct current stimulation. J. Physiol..

[bib34] O’Reilly J.X., Beckmann C.F., Tomassini V., Ramnani N., Johansen-Berg H. (2010). Distinct and overlapping functional zones in the cerebellum defined by resting state functional connectivity. Cereb. Cortex.

[bib35] Parazzini M., Rossi E., Ferrucci R., Liorni I., Priori A., Ravazzani P. (2014). Modelling the electric field and the current density generated by cerebellar transcranial DC stimulation in humans. Clin. Neurophysiol..

[bib36] Petersen S.E., Van Mier H., Fiez J.A., Raichle M.E. (1998). The effects of practice on the functional anatomy of task performance. Proc. Natl. Acad. Sci. USA.

[bib37] Pickering M.J., Garrod S. (2007). Do people use language production to make predictions during comprehension?. Trends Cogn. Sci..

[bib38] Pickering M.J., Garrod S. (2013). An integrated theory of language production and comprehension. Behav. Brain Sci..

[bib39] Pickering M.J., Clark A.W. (2014). Getting ahead: forward models and their place in cognitive architecture. Trends Cogn. Behav..

[bib40] Pope P.A., Pope P.A., Miall R.C. (2012). Task-specific facilitation of cognition by cathodal transcranial direct current stimulation of the cerebellum. Brain Stimul..

[bib41] Pope P.A., Brenton J.W., Miall R.C. (2015). Task-specific facilitation of cognition by anodal transcranial direct current stimulation of the prefrontal cortex. Cereb. Cortex.

[bib42] Ramnani N. (2006). The primate cortico-cerebellar system: anatomy and function. Nat. Rev. Neurosci..

[bib43] Ramnani N. (2011). Frontal lobe and posterior parietal contributions to the cortico-cerebellar system. Cerebellum.

[bib44] Salinas E., Ito M., Romo R. (2008). Control of mental activities by internal models in the cerebellum. Nat. Rev. Neurosci..

[bib45] Schmahmann J.D., Sherman J.C. (1998). The cerebellar cognitive affective syndrome. Brain.

[bib46] Stagg C.J., Jayaram G., Pastor D., Kincses Z.T., Matthews P.M., Johansen-Berg H. (2011). Polarity and timing-dependent effects of transcranial direct current stimulation in explicit motor learning. Neuropsychologia.

[bib47] Stoodley C.J. (2011). Cerebellum and cognition: evidence from functional imaging studies. Cerebellum.

[bib48] Stoodley C.J., Schmahmann J.D. (2009). Functional topography in the human cerebellum: a meta-analysis of neuroimaging studies. Neuroimage.

[bib49] Strick P.L., Dum R.P., Fiez J.A. (2009). Cerebellum and nonmotor function. Annu. Rev. Neurosci..

[bib50] Thach W.T. (1998). What is the role of the cerebellum in motor learning and cognition?. Trends Cogn. Sci..

[bib51] Wolpert D.M., Miall R.C. (2002). Forward models for physiological motor control. Neural Netw..

[bib52] Wolpert D.M., Miall R.C., Kawato M. (1998). Internal models in the cerebellum. Trends Cogn. Sci..

